# Hypoxia Modulates Infection of Epithelial Cells by *Pseudomonas aeruginosa*

**DOI:** 10.1371/journal.pone.0056491

**Published:** 2013-02-13

**Authors:** Bettina Schaible, Siobhán McClean, Andrew Selfridge, Alexis Broquet, Karim Asehnoune, Cormac T. Taylor, Kirsten Schaffer

**Affiliations:** 1 UCD School of Medicine and Medical Science and The Conway Institute, University College Dublin, Dublin, Ireland; 2 School of Pharmacy, Queens's University, Belfast, United Kingdom; 3 Institute of Technology Tallaght, Dublin, Ireland; 4 Laboratoire UPRES EA 3826 «Thérapeutiques cliniques et expérimentales des infections», Faculté de médecine, Université de Nantes, Nantes, France; 5 Centre Hospitalier Universitaire de Nantes, Service anesthésie réanimation chirurgicale, Hôtel Dieu-HME, Nantes, France; 6 Department of Microbiology, St. Vincent's University Hospital, Dublin, Ireland; Duke University Medical Center, United States of America

## Abstract

*Pseudomonas aeruginosa (P. aeruginosa)* is an opportunistic pathogen commonly associated with lung and wound infections. Hypoxia is a frequent feature of the microenvironment of infected tissues which induces the expression of genes associated with innate immunity and inflammation in host cells primarily through the activation of the hypoxia-inducible factor (HIF) and Nuclear factor kappaB (NF-κB) pathways which are regulated by oxygen-dependent prolyl-hydroxylases. Hypoxia also affects virulence and antibiotic resistance in bacterial pathogens. However, less is known about the impact of hypoxia on host-pathogen interactions such as bacterial adhesion and infection. In the current study, we demonstrate that hypoxia decreases the internalization of *P. aeruginosa* into cultured epithelial cells resulting in decreased host cell death. This response can also be elicited by the hydroxylase inhibitor Dimethyloxallyl Glycine (DMOG). Reducing HIF-2α expression or Rho kinase activity diminished the effects of hypoxia on *P. aeruginosa* infection. Furthermore, in an in vivo pneumonia infection model, application of DMOG 48 h before infection with *P. aeruginosa* significantly reduced mortality. Thus, hypoxia reduces *P. aeruginosa* internalization into epithelial cells and pharmacologic manipulation of the host pathways involved may represent new therapeutic targets in the treatment of *P. aeruginosa* infection.

## Introduction

Lower respiratory tract infections are the leading cause of death among infectious diseases. Pulmonary infection with associated intra-alveolar exudates, edematous septal thickening and multiplying pathogens inhibit oxygen diffusion and result in decreased mucosal oxygenation leading to dysregulated gas exchange. *P. aeruginosa* is one of the major pathogens encountered in nosocomial infections causing severe lower respiratory tract infections, skin and soft tissue infections (especially in burn patients) and bacteremia in patients with leukemia, cancer or other immunosuppressive states. In addition *P. aeruginosa* is the main respiratory pathogen encountered in cystic fibrosis where it is associated with increased morbidity and mortality [1]. Hypoxia has been demonstrated in mucus filled airways of cystic fibrosis patients [2]. Treatment of *P. aeruginosa* infections is complicated by rising antimicrobial resistance, absence of an effective vaccine and by the lack of newer antimicrobial agents in development.

Prominent regions of hypoxia are common features of infected and inflamed tissues [3], [4]. In infected tissues, oxygen consumption by bacterial pathogens and phagocytes exacerbates tissue hypoxia. Hypoxia is an important driver of innate immune and inflammatory gene expression in host cells through the activation of transcription factors including Nuclear Factor kappaB (NF-κB) and the Hypoxia inducible factor (HIF) [5], [6], [7]. Furthermore, it has recently become clear that hypoxia can also influence the expression of virulence and antibiotic resistance genes in invading pathogens such as *Shigella* and *Pseudomonas* species respectively [8], [9]. However, despite the recognition that hypoxia independently affects both host and pathogen, less is known about how it impacts upon host-pathogen interactions such as adhesion and infection.

The Hypoxia inducible factor (HIF) is a master regulator of gene expression in metazoan cells exposed to hypoxia [10], [11]. HIF consists of an oxygen-sensitive α-subunit and a constitutively expressed β-subunit. One of three isoforms of the HIF α-subunit bound to a single isoform of the HIF β-subunit constitutes dimeric HIF-1, HIF-2 or HIF-3 respectively [12]. HIF-1 and HIF-2 positively regulate the expression of discreet but overlapping cohorts of genes and demonstrate differential temporal dynamics [13]. HIF-3α is a negative regulator of HIF-1α and HIF-2α [14]. In the presence of sufficient oxygen (normoxia), HIF-α is degraded via hydroxylation by prolyl-hydroxylases (PHD) leading to ubiquitination by the von Hipple Lindau E3 ligase and degradation by the 26S proteasome [12]. The inhibition of the oxygen-dependent prolyl-hydroxylases in hypoxia leads to HIF stabilisation/transactivation with subsequent activation of HIF-dependent target genes. Three PHD isoforms have been identified to date. Among these, normoxic HIF-1α degradation is predominantly regulated by PHD 2. HIF plays a key role in immunity and inflammation by regulating events both in epithelial cells [15] and in immune cells including macrophages, neutrophils, T-cells and dendritic cells [16], [17], [18], [19].

NF-κB consists of a family of transcription factors termed RelA (p65), RelB, c-Rel, p50 and p52 and is a master regulator of inflammation and innate immunity [20]. NF-κB is activated in response to hypoxia both in vitro and in vivo and contributes to the expression of inflammatory genes such as cyclooxygenase-2 (COX-2) [21], [22]. It has recently become appreciated that the same oxygen-sensing hydroxylases that regulate HIF activity in hypoxia also control NF-κB activity during oxygen deprivation [23]. All three PHD isoforms have been implicated in the regulation of both HIF and NF-κB, however PHD2 is the primary isoform involved in the regulation of HIFα stability while PHD1 appears to be the main regulator of hypoxia-dependent NF-κB regulation [21]. Therefore, prolyl-hydroxylases play a central role in the regulation of immune gene expression in hypoxia.

Airway epithelial cells play an important role in host defence. Internalization of *P. aeruginosa* into airway epithelial cells has been demonstrated [24]. The role of bacterial internalization for progression of *P. aeruginosa* infection remains unclear. Here, we have investigated the effects of hypoxia on infection of epithelial cells with *P. aeruginosa* and discovered that activation of specific hypoxia sensitive pathways leads to decreased levels of bacterial internalization which promotes host cell survival. Indeed, it had been reported that activation of the HIF pathway increased epithelial cell barrier function in a *Clostridium difficile* toxin model [25]. A better understanding of the microenvironmental factors effecting host-bacterial interactions may reveal new therapeutic approaches to increasing host resistance against bacterial infection.

## Materials and Methods

### Ethics Statement

Mice used for in vivo experiments were treated in accordance with institutional policies and the guidelines stipulated by the animal welfare committee. The Committee of Animal Ethics of the University of Nantes approved all animal experimentation in this study.

### Bacterial and cell culture

The *P. aeruginosa* strain ATCC 27853 and *E. coli* strain NCIB 9485 were purchased from ATCC (Manassus, VA). Clinical isolates used were derived from the sputa of cystic fibrosis patients chronically infected with *P. aeruginosa*. Bacteria were grown at 37°C on LB agar plates (Sigma-Aldrich, Dorset UK) or in liquid broths (Müller Hinton broth II, BD microbiology systems, Cockeysville MD) at 21% or 1% atmospheric oxygen. For heat-inactivation, bacteria in suspension were quantified and heat killed by exposure to 65°C for 2 h. To ensure that no living bacteria survived, 10 µl of the suspension was cultured on a LB agar plate and incubated overnight at 37°C.

A549 cells were grown in DMEM F12 Ham media (Sigma-Aldrich, Dorset UK) with 10% foetal calf serum (Gibco, Paisley, UK), 2 mM L-glutamine (Gibco, Paisley, UK), 100 U/ml penicillin and 100 µg/ml streptomycin (Gibco, Paisley, UK). Caco2 and MEF cells were cultured in DMEM media (Gibco, Paisley, UK) with 1% foetal calf serum, 100 U/ml penicillin and 100 µg/ml streptomycin. HepG2 wild type and cells stably transfected with shRNA sequences against HIF-1α and HIF-2α were a gift from Prof. B. Brüne (University of Frankfurt). Media for stably transfected HepG2 cells additionally contained 2 µg/ml puromycin dihydrochloride (Sigma-Aldrich, Dorset UK). 16HBE 14o- [26] and CFBE41o- cells (homozygous for ΔF508 mutation of the CFTR gene) were a gift of Prof. D. Gruenert (University of California, San Francisco) and were cultured in MEM with 10% FCS 2 mM L-glutamine and 100 U/ml penicillin/100 µg/ml streptomycin on a collagen/fibronectin/BSA coated surface.

Normoxic controls were cultured in 21% oxygen and 5% CO_2_ in a humidified environment at 37°C. For culture in hypoxia, cells were transferred to a hypoxia chamber (Coy Laboratories, Grass Lake, MI; Ruskin Technologies, Leeds, U.K.) with a humidified atmosphere of 1% oxygen, 5% CO_2_ and the balance N_2_ at 37°C. At the onset of hypoxic exposure, the cell culture media was replaced with pre-equilibrated medium ensuring that the cells experienced an instantaneous drop in pO_2_ levels.

### Drugs and Reagents

To activate different TLR pathways, the following specific agonists were used; flagellin (from *S. thymimurium* 0.5 µg/ml, InvivoGen, San Diego, CA) for TLR5, Pam_3_CysSK_4_ (2 µg/ml, ECM microcollections, Tübingen, Germany) for TLR2 or LPS (*P. aeruginosa* derived, 0.5 µg/ml, Sigma-Aldrich, Dorset, UK) for TLR4. TNFα (10 ng/ml, Sigma-Aldrich, Dorset, UK) served as positive control. To mimic hypoxia, the pan-hydroxylase inhibitor Dimethyloxallyl Glycine (DMOG, Cayman Chemicals, Ann Arbor, MI) was used at 1 mM. Y27632 (Sigma-Aldrich, Dorset, UK) inhibited selective ROCK (Rho-associated coiled-coil forming protein serine/threonine kinase) at 10 µM. Inhibition of the NF-κB pathway was achieved by using Bay 11-7082 (Merck, Darmstadt Germany) at 10 µM, an inhibitor of IκBα phosphorylation. Controls were treated with the appropriate vehicle control.

### Adhesion and Internalization Assays

A549 cells, MEF cells, 16HBE14o- and CFBE410- cells were seeded at 2×10^5^ per well in 24 well plates (HepG2 cells at 6×10^5^ per well). Before bacterial infection, cells were pre-incubated for 24 h in normoxia or hypoxia, in antibiotic free culture media pre-equilibrated to 1% or 21% oxygen respectively. Chemical inhibitors were added at the same time.

Bacteria were grown to mid log phase at an OD_600_ of 0.5–0.6. A549 cells and MEF cells were infected with bacteria at a MOI of 50∶1 (or 16∶1 for HepG2 cells). After incubation of infected cells for four hours (or indicated incubation time) under normoxic or hypoxic conditions, the medium was replaced with fresh medium containing 1 mg/ml amikacin (Sigma-Aldrich, Dorset UK) and 1 mg/ml ceftazidime (Sigma-Aldrich, Dorset UK) and cells were incubated for further 2 h in the presence of antibiotics to kill extracellular bacteria. Amikacin concentrations were increased to 4 mg/ml for experiments with clinical strains in hypoxia to reliably kill all extracellular bacteria. Inhibitors were added at every step during the experiment where media was replaced to maintain a constant concentration. After 2 h incubation in the presence of antibiotics, the media was removed and cells were washed three times with PBS. Fluid from the last washing step was plated on agar plates to document the absence of viable extracellular bacteria. After washing, 500 µl of cell-lysis buffer (PBS, 10 mM EDTA, 0.025%TritonX 100) was added for 20 min. The remainder of attached cells were scraped of the well and the lysate was plated on LB agar plates in duplicate. CFUs were counted after 24 h to 48 h incubation at 37°C.

To measure the number of adherent bacteria, cells were infected as for internalisation assays without the use of antibiotics to kill extracellular bacteria. After washing 3 times, cells were lysed as described above. The lysate, containing all cell associated bacteria (adherent and internalised), was plated on LB agar plates and quantified after 24 h incubation at 37°C. The number of adherent bacteria was calculated as the number of associated bacteria minus the number of internalized bacteria.

### Lactate Dehydrogenase (LDH) assays

Cytotoxicity was determined by quantifying LDH released into cell culture media (Cytotoxicity Detection Kit, LDH, Roche, Dublin Ireland) via enzyme activity of LDH. After bacterial infection the complete cell culture media was removed for quantification of LDH released into the medium. To quantify total LDH, cells were lysed with Triton X (1% in serum free cell culture media) overnight at 4°C. 50 µl media or lysate were transferred with 50 µl of reaction mix in a 96 well plate and incubated at RT for 30 minutes in the dark. Absorbance was measured at 490 nm. Toxicity was calculated as LDH released into the medium as percentage of total LDH present in cells.

### Western blotting and luciferase assay

Whole cell lysates were prepared as previously described [27], [28]. The following antibodies were used for western blotting: p65, COX-2 (Santa Cruz Biotechnology. Santa Cruz, CA), HIF-1α (BD Transduction Laboratories, Oxford, UK), HIF-2α (Novus, St. Charles, MO) and β-actin (Sigma-Aldrich, Dorset, UK).

For luciferase reporter assays A549 cells were transfected with 125 ng pGL4.32[*luc2P*/NF-κB-RE/Hygro] luciferase reporter construct (Promega, Madison WI) and 25 ng pSV-β galactosidase vector (Promega, Madison WI). The following day the cells were pre-incubated for 24 h in either 21% oxygen or 1% oxygen and subsequently stimulated with specific ligands for TLR2, TLR4 and TLR5 for 6 h in the indicated oxygen concentration. Cells were washed with PBS and lysed with lysis buffer (Promega, Madison WI). Luciferase activity of the lysate was determined after adding luciferase substrate (Promega, Madison WI) by luminometry (Berthold Huntsville, AL).

### Mouse pneumonia model

Six–week-old pathogen-free RjOrl∶SWISS mice (CD-1 mice, weight 20–24 g) were purchased from Janvier Laboratories (Le Genest Saint Isle, France). Mice were maintained on a 12-hour light/dark cycle with access to food and water ad libitum. *P. aeruginosa* strain PAO1 was grown overnight in Brain Heart Infusion medium at 37°C under agitation. Immediately before use, the bacterial pellet (centrifuged at 1,000 g for 10 min) was washed twice using 0.9% NaCl. After the second wash, the pellet was resuspended in sterile saline buffer and the inoculum was calibrated by nephelometry (2×10^8^ CFU/ml).

Mice were assigned to four groups (n = 10 per group): pneumonia alone (control group), DMOG treatment (8 mg/mouse, intraperitoneally) at the time of infection with *P. aeruginosa*, DMOG treatment 24 hours prior to infection and DMOG treatment 48 hours prior to infection. Pneumonia was induced as previously described [29]. Briefly, mice were anaesthetized with sevoflurane (Abbott, Chicago, IL, USA) and placed in dorsal recumbency. Transtracheal insertion of a 24-gauge feeding needle was used to inject 75 µl of a bacterial suspension adjusted to 2×10^8^ CFU/ml (1.5×10^7^ CFU/mouse). Survival was assessed twice per day up to 6 days following infection.

### Statistical analysis

Data are presented as mean ± SEM for n independent experiments. Statistical analysis was carried out using unpaired student's t-test (Microsoft Excel 2010) or one-way analysis of variance ANOVA analysis with Tukey post test (Graph pad Prism 5 Software). Densitometry analysis was performed with Image J Software. Statistical significance was denoted as p<0.05. Hazard ratio for survival in the treated animal group versus the untreated group was calculated using GraphPad Prism software (San Diego, USA).

## Results

### Hypoxia decreases epithelial cell infection with *P. aeruginosa*

**Figure 1:**
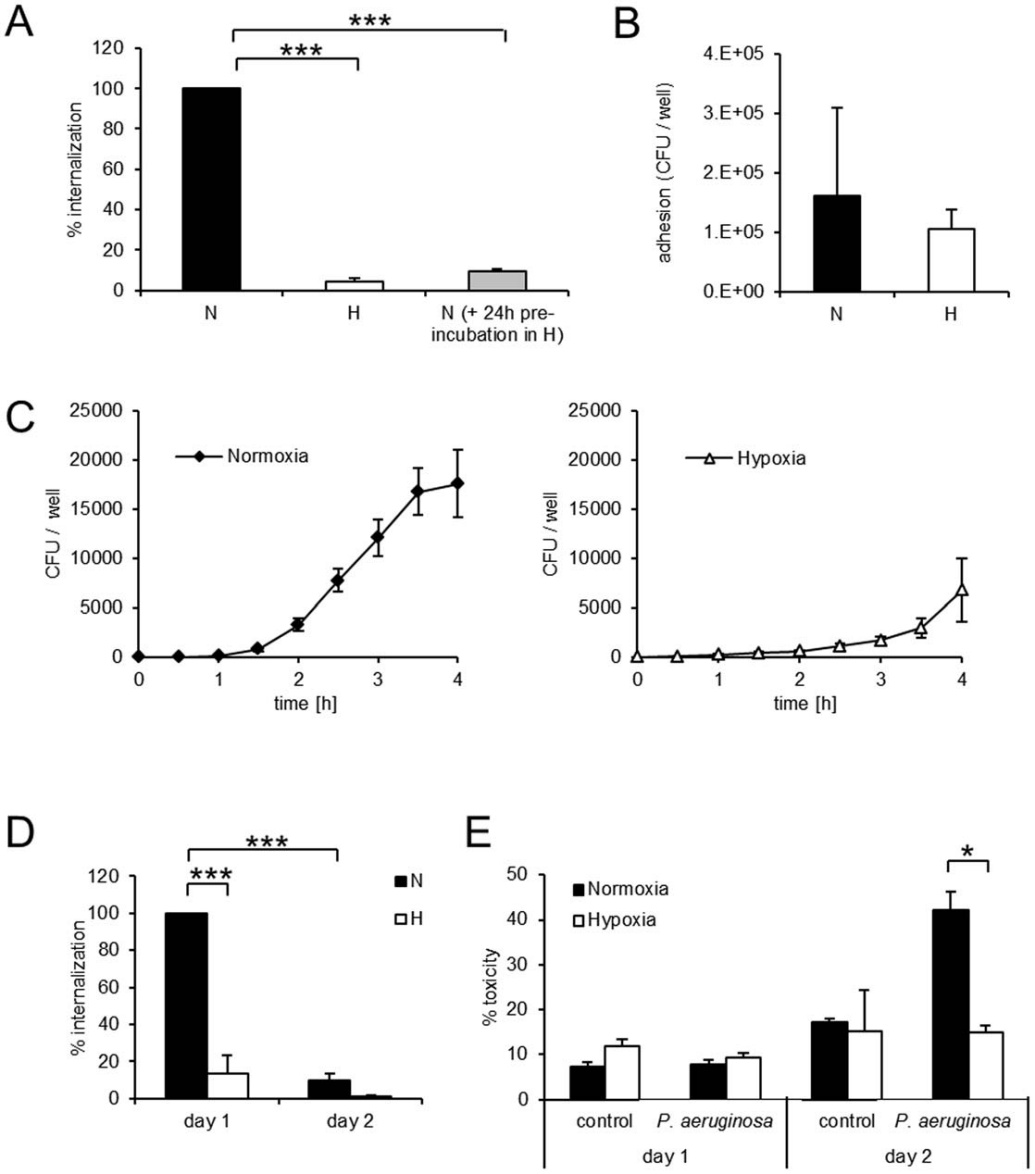
Hypoxia prevents internalization of *P. aeruginosa* into epithelial cells. A549 cells were infected with *P. aeruginosa* under normoxic (N) and hypoxic (H) conditions, after pre-incubation for 24 h in the respective environment for all experiments (A–E). A: Internalization of *P. aeruginosa* in normoxia, hypoxia and in normoxia after pre-incubation in hypoxia. B: Adhesion of *P. aeruginosa* in normoxia and hypoxia. C: Uptake of *P. aeruginosa* over time in normoxia and hypoxia. D: Internalization of *P. aeruginosa* directly after infection (day 1) or 24 h after infection (day 2). E: Cell toxicity of uninfected and *P. aeruginosa* infected cells was measured by LDH release directly after infection (day 1) or 24 h after infection (day 2). Data represent mean ± SEM of 3 independent experiments throughout (*<p 0.05; *** p<0.0001).

Adhesion and internalization are distinct events in the encounter between bacterial pathogens and host epithelial cells. Using antibiotic exclusion assay, we found that the internalization of *P. aeruginosa* greatly exceeds that of *Escherichia coli (E. coli)*, highlighting the invasive potential of *P. aeruginosa* for pulmonary epithelial cells (Figure S1). Furthermore, internalization of *P. aeruginosa* into A549 cells was decreased by 94.7±1.6% in hypoxia compared to normoxia (Figure 1A). Notably, a similar decrease in internalization was observed in epithelial cells which had been pre-incubated in hypoxia for 24 h (Figure 1A), indicating that the effects of hypoxia on infection are due for the most part to changes in host epithelial cells.

There was no difference in levels of adherence of *P. aeruginosa* to epithelial cells in hypoxia compared to normoxia (Figure 1B) demonstrating that decreased internalization is not a result of decreased bacterial adhesion.

The decreased numbers of internalized bacteria in hypoxia could be a result of decreased bacterial uptake into the host cell or increased intracellular killing. To determine which is the case, we performed a time course experiment assessing intracellular bacterial numbers at 30 minutes intervals following infection. In hypoxia, the number of internalized *P. aeruginosa* was decreased at all-time points from 1 to 4 hours post infection compared to normoxia (Figure 1C). We concluded that hypoxia decreased internalization rather than increased intracellular killing, as for increased intracellular killing we would have expected equal initial numbers of internalized bacteria in normoxia and hypoxia followed by a faster rate of decline in hypoxia. 24 h following infection the number of internalized bacteria had decreased from 100% to 9.6% in normoxia and from 13.8% to 1.5% in hypoxia (Figure 1D).

Next, we investigated the consequence of decreased bacterial uptake in hypoxia for host cell survival. *P. aeruginosa* is cytotoxic and capable of inducing death in epithelial cells [30]. To assess cytotoxicity in our model, we measured Lactate dehydrogenase (LDH) release following bacterial infection. 24 hours following infection, normoxic *P. aeruginosa* infected cells released significantly more LDH than hypoxic *P. aeruginosa* infected cells indicating decreased induction of cell death by *P. aeruginosa* infection under hypoxic conditions (Figure 1E). These data support a protective role for hypoxia in reducing epithelial cell infection by *P. aeruginosa*.

### DMOG mimics the effects of hypoxia on *P. aeruginosa* infection of epithelial cells

**Figure 2:**
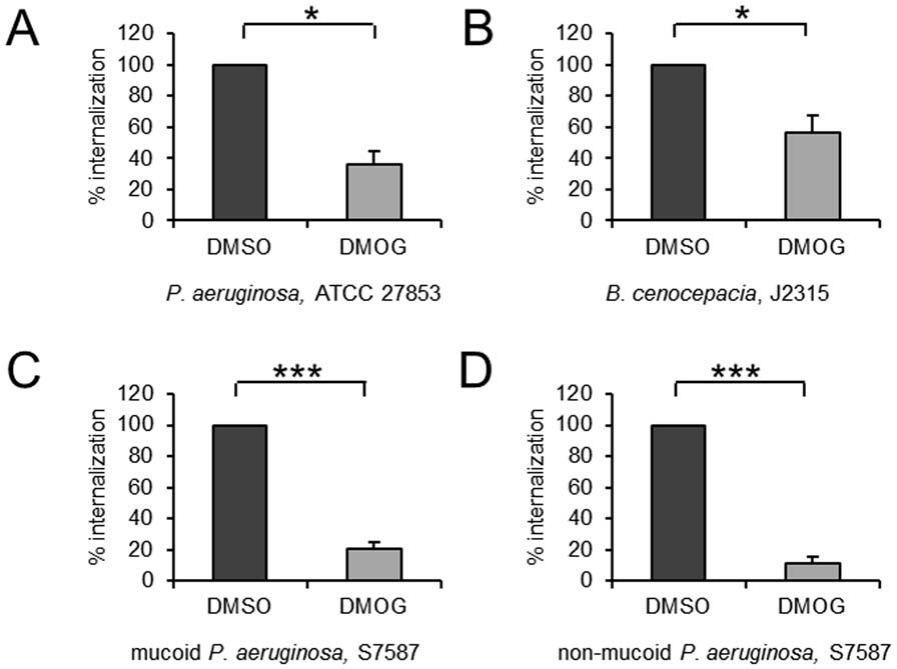
Bacterial internalization is decreased in DMOG treated cells. To mimic hypoxia, A549 cells were pre-treated with 1 mM of the pan-hydroxylase inhibitor DMOG or its' solvent DMSO for 24 h and throughout following infection with the *P. aeruginosa* reference strain ATCC 27853 (A), a CF pathogen *B. cenocepacia*, *J2315* (B) and two clinical *P. aeruginosa* isolates from CF patients, S7587, mucoid and non-mucoid *P. aeruginosa* (C and D). Data represent mean ± SEM of 3 independent experiments throughout (* p<0.05; *** p<0.0001).

To investigate the therapeutic potential of manipulating hypoxia sensitive pathways in *P. aeruginosa* infection, we tested the effect of the hydroxylase inhibitor DMOG on *P. aeruginosa* infection of epithelial cells. Because phenotypic differences in bacterial invasion and intracellular replication have been described for various *P. aeruginosa* strains, we investigated the impact of DMOG on the uptake of multiple strains. We used mucoid and non-mucoid *P. aeruginosa* isolates which we cultured from the sputa of cystic fibrosis patients. We also tested the effect of DMOG on uptake of *Burkholderia cenocepacia* (*B. cenocepacia*), an invasive pathogen also found in cystic fibrosis patients. We verified that bacterial multiplication took place in the presence of DMOG. Internalization of all *P. aeruginosa* strains and *B. cenocepacia* were significantly decreased in cells pre-treated with DMOG, indicating that mimicking hypoxia with pharmacologic hydroxylase inhibition may be a novel approach to preventing infection of pulmonary epithelial cells by *P. aeruginosa* and *B. cenocepacia* (Figure 2A–D).

### Hypoxia amplifies *P. aeruginosa*-induced NF-κB acitivity but this does not account for decreased *P. aeruginosa* internalization

**Figure 3:**
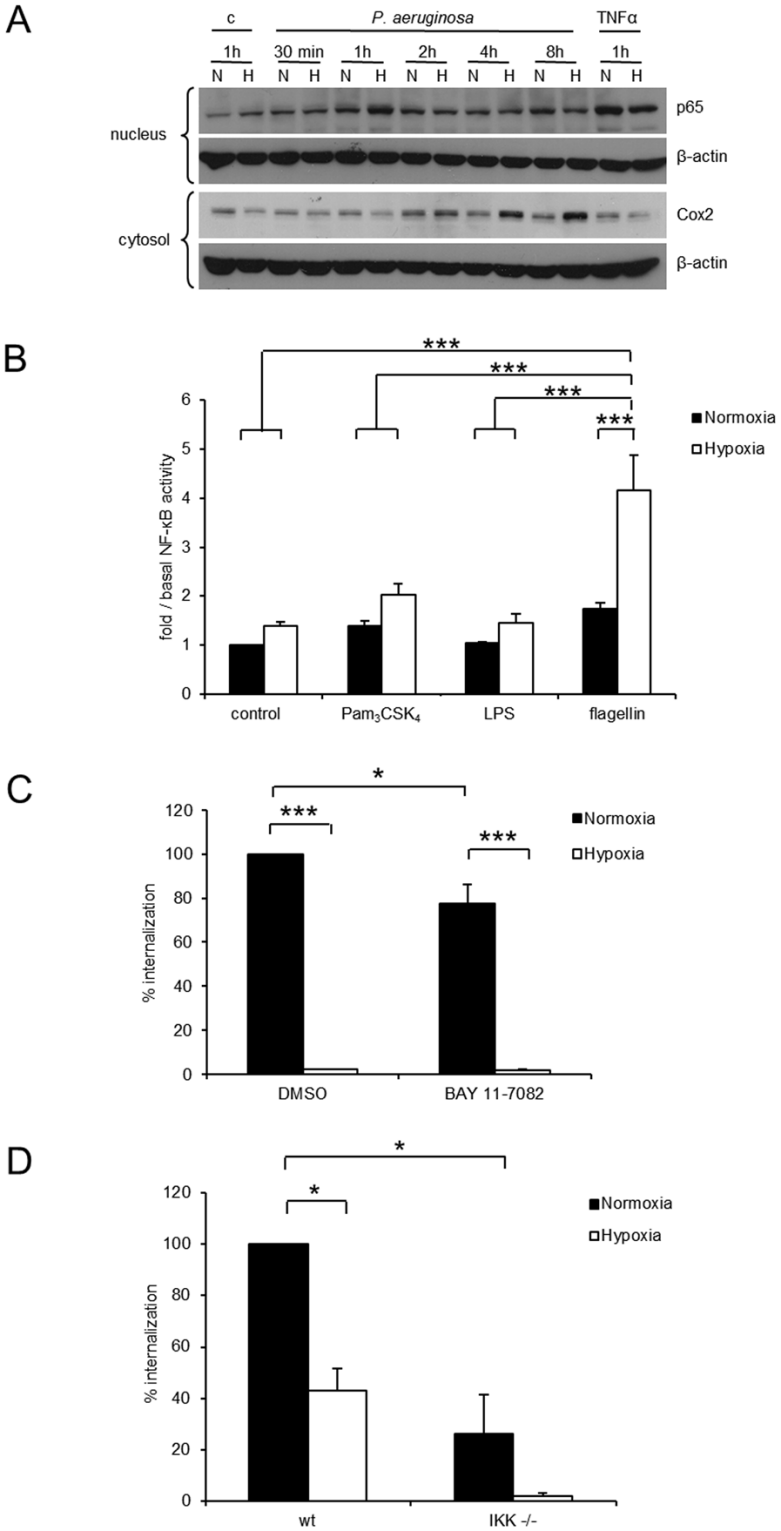
Hypoxia increases NF-κB activity in response to *P. aeruginosa* but silencing of NF-κB fails to normalize hypoxia induced decreased internalization. A: Protein levels of nuclear p65 and cytosolic Cox2 were investigated by immunoblot in A549 cells stimulated with heat inactivated *P. aeruginosa* in normoxia (N) and hypoxia (H) for the indicated times. B: NF-κB transcription factor activity was measured by a NF-κB luciferase reporter assay in normoxic and hypoxic A549 cells stimulated with TLR ligands for TLR2 (Pam3CK4, 2 µg/ml), TLR4 (LPS, 0.5 µg/ml) and TLR5 (flagellin, 0.5 µg/ml). Data represent mean ± SEM of 6 independent experiments (*** p<0.0001). C: Intracellular *P. aeruginosa* in A549 cells in normoxia or hypoxia in the presence or absence of the NF-κB inhibitor BAY 11-7082 (10 µM) or its solvent DMSO were determined in an antibiotic protection assay. Data represent mean ± SEM of 4 independent experiments (* p<0.05, *** p<0.0001). D: Antibiotic protection assay with *P. aeruginosa* in MEF wt and IKK−/−cells under normoxic and hypoxic conditions. Data represent mean ± SEM of 2 independent experiments (* p<0.05).

We next investigated potential mechanisms underpinning the effects of hypoxia on bacterial uptake into pulmonary epithelial cells. Because NF-κB is the master regulator of host innate immune responses to bacterial pathogens and up-regulation of NF-κB activity is associated with enhanced *P. aeruginosa* clearance from the lungs [31], we investigated whether hypoxia altered NF-κB activity in *P. aeruginosa* infected cells. Hypoxia amplified nuclear localization of NF-κB in *P. aeruginosa* infected pulmonary and colonic epithelial cells resulting in increased expression of the NF-κB target cyclooxygenase 2 (COX-2) (Figure 3A+S2).

To investigate whether hypoxia elicited increased NF-κB activity is equally mediated by all three major toll like receptors (TLRs), NF-κB-dependent luciferase reporter assays with selective TLR 2, 4 and 5 agonists were performed. In hypoxia, stimulation of TLR 5 by flagellin resulted in significantly higher NF-κB activity than stimulation of TLR 2 by Pam_3_CysSK_4_ or stimulation of TLR 4 by LPS (Figure 3B). We therefore concluded that TLR 5 mediated signalling is mainly responsible for increased nuclear translocation of p65.

We next investigated whether amplified NF-κB activity contributed to decreased bacterial internalization in hypoxia using two experimental approaches. First, bacterial internalization assays were performed in the presence of the NF-κB inhibitor BAY 11-7082. While BAY 11-7082 decreased *P. aeruginosa* internalization in normoxia, it did not impact upon hypoxia-induced decreases in internalization (Figure 3C). Second, to be able to investigate bacterial internalization in the presence of a complete knockdown of NF-κB, we performed internalization assays with IKK knockout mouse embryonic fibroblasts. *P. aeruginosa* internalization into mouse embryonic fibroblasts had been shown before [32]. Internalization of *P. aeruginosa* was diminished in IKK knockout cells and reversal of hypoxia-induced effects was not observed (Figure 3D). Thus, while hypoxia amplifies *P. aeruginosa* induced NF-κB activity, inhibiting NF-κB activity in hypoxia did not impact on decreased bacterial uptake indicating that the effects of hypoxia on *P. aeruginosa* uptake are independent of NF-κB.

### HIF-2α partially mediates decreased bacterial internalization in hypoxia

**Figure 4:**
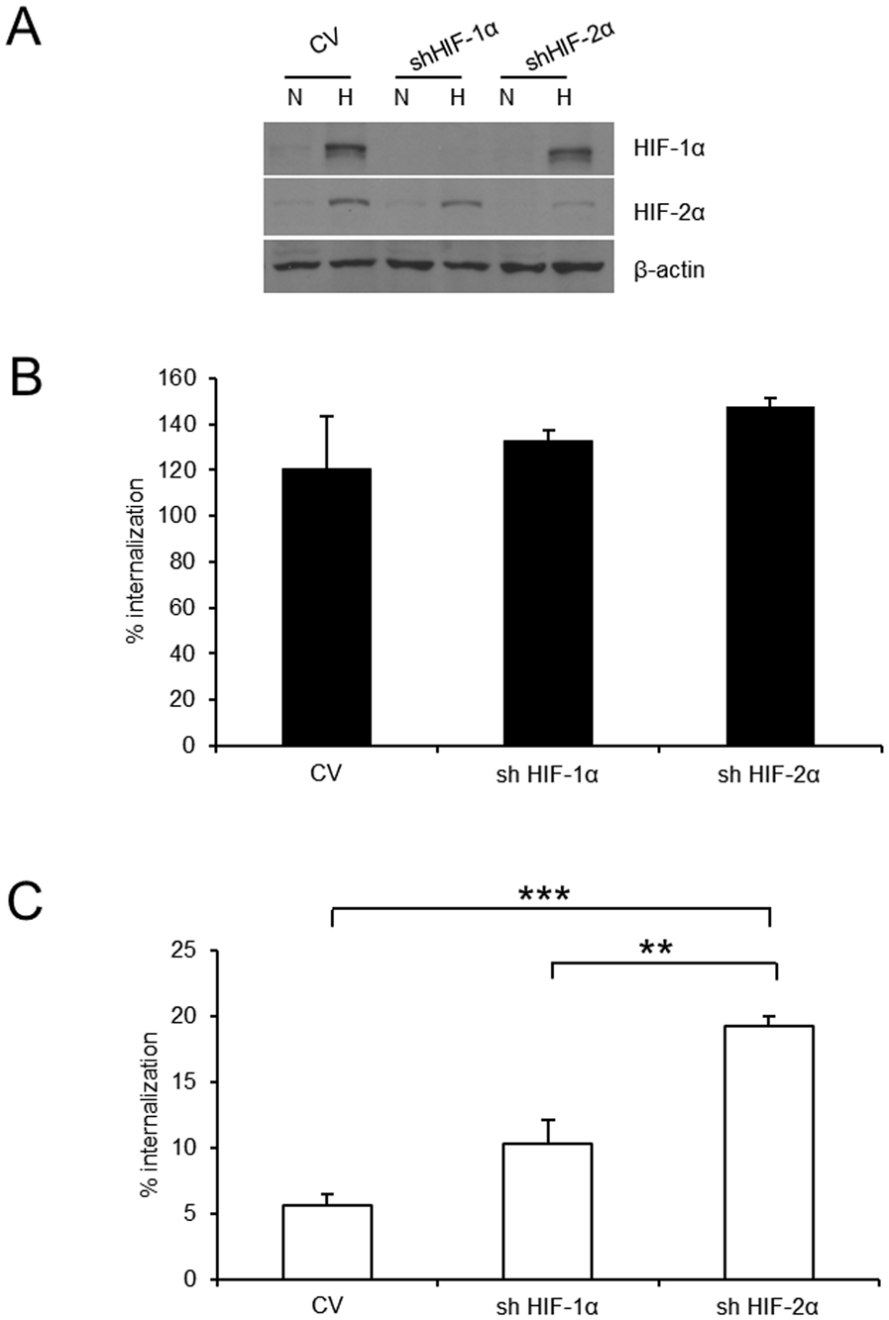
HIF-2α is partly responsible for decreased internalization of *P. aeruginosa* in hypoxia. A: Knock down of HIF-1α and HIF-2α protein in HepG2 cells stably expressing shRNA directed against HIF-1α and HIF-2α shown by immunoblot. B: Antibiotic protection assay in normoxia with *P. aeruginosa* in HepG2 cells (see A). Internalized *P. aeruginosa* are shown as % of normoxic control. Data represent mean ± SEM of 3 independent experiments. C: Antibiotic protection assay in hypoxia with *P. aeruginosa* in HepG2 cells (see A). Internalized *P. aeruginosa* are shown as % of normoxic control. Data represent mean ± SEM of 3 independent experiments (** p<0.01, *** p<0.0001).

We next investigated whether increased HIF activity may play a role in the decreased internalization of *P. aeruginosa* observed in hypoxia. Activation of HIF has been demonstrated for a variety of bacterial infections [33]. We tested the effects of depletion of HIF-1α or HIF-2α using HepG2 cells stably transfected with short hairpin plasmids. Analysis by densitometry revealed an 89% knockdown of HIF-1α expression and 79% knockdown of HIF-2α (Figure 4A). Bacterial internalization in normoxia was not affected by silencing HIF-1α or HIF-2α respectively (Figure 4B). Bacterial internalization into hypoxic control vector epithelial cells was reduced to 5.6%±0.8% of normoxic epithelial cells, whereas bacterial internalization into hypoxic HIF-2α knockdown cells was only reduced to 19.3%±0.7% of normoxic controls (Figure 4C) indicating that HIF-2α was participating in the hypoxia elicited effect.

### Hypoxia enhances RhoA activity in *P. aeruginosa* infected cells and inhibition of this pathway partially normalizes decreased bacterial internalization

**Figure 5:**
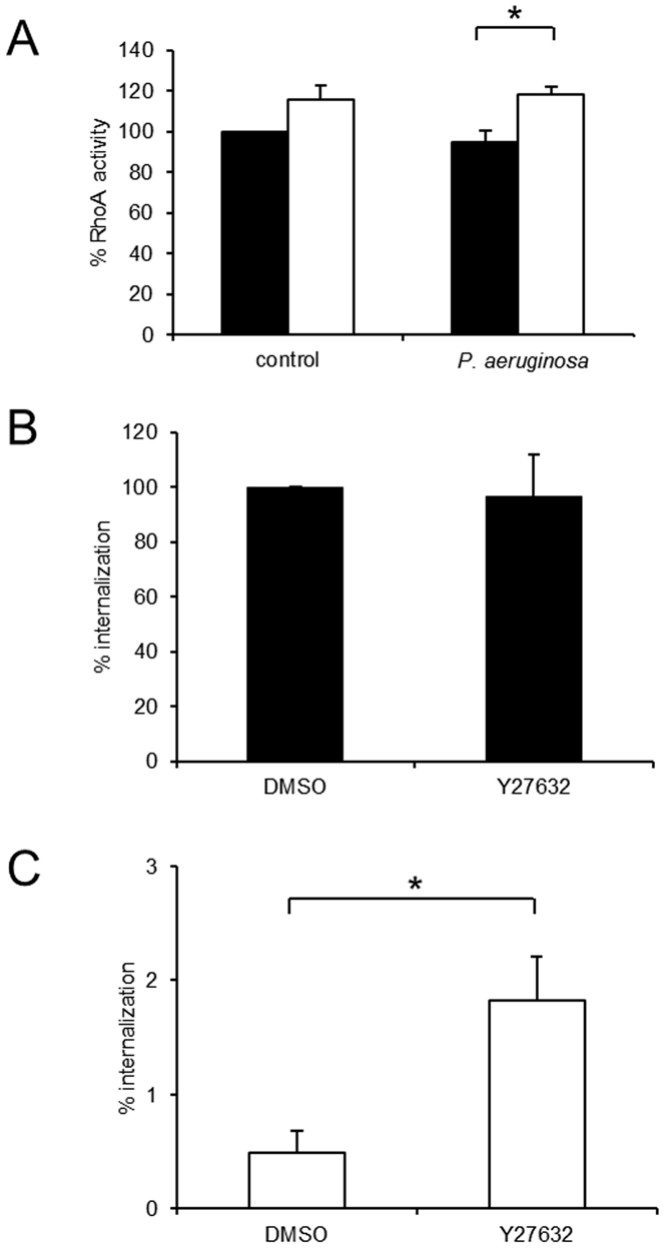
Increased RhoA activity in hypoxic, *P. aeruginosa* infected cells is partly responsible for decreased internalization of *P. aeruginosa* in hypoxia. A: Active RhoA (RhoA-GTP) was determined by ELISA in A549 cells pre-incubated in normoxia and hypoxia followed by infection with *P. aeruginosa*. Data represent mean ± SEM of 3 individual experiments (* p<0.05). B: Normoxic A549 cells were pre-treated with 10 µM Y27632 and throughout the following infection with *P. aeruginosa*. Internalized *P. aeruginosa* as % of normoxic control are shown. Data represent mean ± SEM of 5 individual experiments. C: Intracellular *P. aeruginosa* in A549 cells were determined in hypoxia with the same treatments as described in B. Internalized *P. aeruginosa* as % of normoxic control are shown. Data represent mean ± SEM of 5 individual experiments (* p<0.05).

*P. aeruginosa* entry into cells is an actin-dependent process involving actin rearrangement regulated by Rho GTPases. Furthermore, *P. aeruginosa* exotoxins ExoS and ExoT have been demonstrated to affect Rho GTPase activity and inhibit bacterial internalization [34]. RhoA is a key regulator of the cytoskeleton of eukaryotic cells. RhoA activity increased in A549 cells in the presence of hypoxia upon infection with *P. aeruginosa* (Figure 5A).

Increased Rho activity leads to activation of downstream serine/threonine kinases (ROCK family). To determine the importance of Rho activity for decreased internalization in hypoxia, we performed internalization experiments in the presence of Y27632, a ROCK inhibitor [35]. Bacterial internalization in normoxia remained unchanged in the presence of Y27632, whereas Y27632 partially inhibited the reduced internalization observed in hypoxia (Figure 5B and 5C).

Stress fibre formation as a marker of cytoskeleton re-arrangement was increased in hypoxia and abolished by the ROCK inhibitor Y 27632 (Figure S3). Whereas hypoxia induced stress fibre formation was not attenuated in HIF-1α knockdown cells, stress fibre formation was decreased in hypoxia in HIF-2α knockdown cells suggesting different roles for the two HIF-α isoforms in cytoskeleton rearrangement (Figure S3).

### Hypoxia decreases *P. aeruginosa* internalization independent of CFTR expression

**Figure 6:**
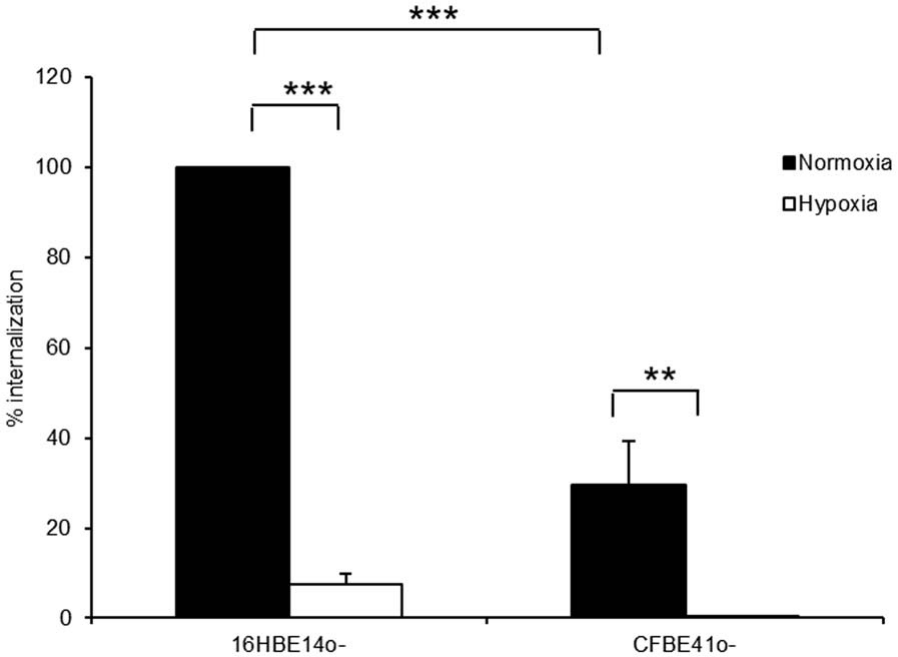
Decrease of intracellular *P. aeruginosa* in hypoxia is independent of CFTR. Intracellular *P. aeruginosa* were determined with an antibiotic exclusion assay in 16HBE14o- and CFBE41o- cells under normoxic and hypoxic conditions. Data represent mean ± SEM of 5 individual experiments (** p<0.01; *** p<0.0001).

The cystic fibrosis transmembrane conductance regulator protein (CFTR) has been postulated as an important regulator of pulmonary epithelial cell internalization of *P. aeruginosa* and the absence of a functional CFTR protein was associated with decreased *P. aeruginosa* internalization [36], [37]. Decreased expression of CFTR in response to hypoxia has been observed in intestinal epithelial cells [38]. To investigate whether decreased *P. aeruginosa* internalization in hypoxia is dependent upon a functional CFTR, we investigated bacterial uptake in the bronchoepithelial cell line 16HBE 14o- and CFBE41o- cells (homozygous for ΔF508 mutation of the CFTR gene). Internalization of *P. aeruginosa* decreased to 29.7±9.6% of internalization into 16HBE 14o- cells in the absence of functional CFTR protein in CFBE41o-cells under normoxic conditions (Figure 6). Despite the overall decrease in internalized bacteria, the magnitude of the hypoxia elicited decreased internalization was comparable in the two cell lines: From 100% to 7.4±2.5% in 16HBE 14o- and from 100% to 1.3±0.6% in CFBE41o- cells, indicating that hypoxia decreases bacterial internalization independent of the presence of a functional CFTR (Figure 6).

### DMOG decreases mortality in vivo in a pneumonia infection model

**Figure 7:**
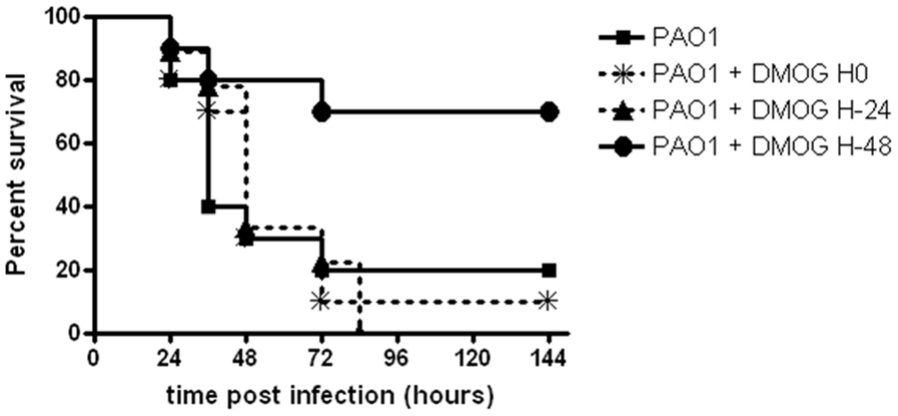
DMOG improves survival in a murine *P. aeruginosa* pneumonia model. Pneumonia was induced by instillation of 1.5×10^7^ CFU/mouse of *P. aeruginosa.* Mice were assigned to four groups (n = 10 per group): pneumonia alone (control group), DMOG treatment (8 mg/mouse, intraperitoneally) at the time of infection with *P. aeruginosa*, DMOG treatment 24 hours prior to infection and DMOG treatment 48 hours prior to infection. DMOG treatment 48 h before infection significantly increased survival of mice with a mortality of only 20% at 48 hpi and a final mortality rate of 30% compared to 80–100% in the other groups.

We next investigated whether the effects of prolyl-hydroxylase inhibition on bacterial internalization observed in vitro would impact on the course of infection in vivo. Mice were infected intratracheally with *P. aeruginosa*. DMOG was injected 48 h or 24 h before infection or simultaneous with *P. aeruginosa* infection. DMOG treatment on day 0 or 24 h before infection did not affect the survival rate of mice infected with *P. aeruginosa*. In contrast DMOG treatment 48 h before infection significantly increased survival of mice with a mortality of only 20% at 48 hpi and a final mortality rate of 30% compared to 80–100% in the other groups (hazard ratio for survival 3.533; 95% CI 1.185–16.81; p = 0.0270) (Figure 7).

## Discussion

Epithelial cells are the first barrier encountered by bacterial pathogens and the interactions between bacteria and human epithelial cells are fundamental for the pathogenesis of bacterial infections. In bacterial infections, influx of inflammatory cells and multiplication of pathogens increases oxygen demands and hypoxia is subsequently a frequently encountered component of inflammatory environments in which infections take place. *P. aeruginosa* is the leading gram-negative pathogen in ventilator-associated pneumonia [39]. Intensive care mortality rates are significantly higher for bacteremic pneumonia compared to non-bacteremic pneumonia [40]. In the current study, we demonstrate that hypoxia strikingly attenuates internalisation of the human opportunistic pathogen *P. aeruginosa* into epithelial cells, potentially conferring increased resistance against bacterial invasion to epithelial cells. Hypoxia-induced decreases in internalization were not only observed in A549 pulmonary epithelial cells, but equally in 16 HBE 14o- human bronchoepithelial cells, an immortalized but non-transformed cell line retaining differentiated epithelial morphology and functions [26].

Despite phenotypic differences in invasion and intracellular replication among *P. aeruginosa* strains, all strains of *P. aeruginosa* were found to be capable of entering host cells in vitro [41], [42]. Similarly in vivo, alveolar epithelial cells II were found to harbour intracellular *P. aeruginosa* in a murine infection model [43]. The pathophysiological consequences of *P. aeruginosa* internalisation into airway epithelial cells are unclear.

In this study LDH release was significantly increased in normoxic cells 24 h after infection compared to hypoxic cells. Invasive *P. aeruginosa* isolates were found to penetrate epithelial cell monolayers by either evoking epithelial cell death with subsequent disruption of monolayers or by transmigrating through epithelial cells [30]. Increased internalization resistance in response to hypoxia could therefore confer a cytoprotective effect upon epithelial cells and decrease transmigration of invasive bacteria. This is supported by our observation in vivo.

Hypoxia elicited decreased internalization was not restricted to the ATCC 27853 control strain, as two clinical *P. aeruginosa* isolates and a *B. cenocepacia* reference strain were equally impaired in their potential to internalize into pulmonary epithelial cells. Cystic fibrosis *P. aeruginosa* isolates have been found to attenuate virulence factor expression as part of immune evasion strategies in chronic infection [44]. Therefore it was not surprising that the absolute number of internalized bacteria was lower for the two CF isolates in comparison to the ATCC strain. Despite decreased numbers of internalized bacteria in normoxia, the relative decrease elicited by hypoxia was comparable for all clinical isolates, suggesting that rather than being a bacterial strain specific effect, decreased internalization was a host cell attributable phenomenon.

Two additional experimental observations suggested that hypoxia elicited epithelial cell responses are the major contributor to decreased bacterial internalization in hypoxia: 1. Pre-exposure of epithelial cells to hypoxia increased epithelial cell resistance to subsequent bacterial infection. 2. Applying Dimethyloxallyl Glycine (DMOG) decreased bacterial internalization. DMOG inhibits prolyl-hydroxylation of HIF-α subunits leading to increased HIF-α protein stability and increased transcriptional activity [45].

Epithelial cell signalling events elicited by bacterial internalization remain unclear. The two major transcription factors controlling hypoxia driven epithelial cell responses are NF-κB and HIF. Blocking NF-κB activity did not reverse decreased bacterial internalization in hypoxia. Increased HIF activity had been demonstrated for a variety of bacterial infections [33]. Although silencing of HIF-1α did not impact on bacterial internalization in hypoxia, silencing of HIF-2α partially restored bacterial internalization.

The inhibition of bacterial internalization by DMOG suggested that prolyl- hydroxylases would play an important part in the observed effect. Knockdown of prolyl-hydroxylases in HeLa cells resulted in activation of RhoA [46] and RhoA had been shown to be induced by hypoxia in mouse embryonic fibroblasts [47] and in renal cancer cells [48]. RhoA activity increased in pulmonary epithelial cells in the presence of hypoxia upon infection with *P. aeruginosa*. Inhibition of RhoA activity using an inhibitor partially restored *P. aeruginosa* internalization in hypoxia.

The cystic fibrosis transmembrane conductance regulator (CFTR) had been reported to function as an epithelial cell receptor facilitating internalization of *P. aeruginosa* into respiratory epithelial cells [37]. In pulmonary and intestinal epithelial cells, HIF has been reported to decrease CFTR expression [38], [49], whereas hypoxia increased CFTR expression in corneal epithelial cells leading to increased *P. aeruginosa* binding and internalization [50]. Although bacterial internalization into bronchoepithelial cells was decreased, hypoxia still diminished bacterial internalization in the absence of functional CFTR suggesting that CFTR did not play a role in mediating decreased internalization in hypoxia. In the context of cystic fibrosis airway disease, absence of functional CFTR protein and presence of hypoxia in mucus filled airways could exert additive effects leading to complete inhibition of *P. aeruginosa* internalization.

In vivo HIF-1α deletion resulted in more severe *Clostridium difficile* toxin induced intestinal injury and inflammation [25]. In response to activation of the HIF pathway epithelial cells showed increased mucosal barrier function through activation of intestinal trefoil factor [51] and increased production of anti-inflammatory signalling molecules such as adenosine [52]. In the murine pneumonia model used in this study DMOG significantly decreased mortality in infected mice when given 48 h before *P. aeruginosa* infection. In contrast HIF increased translocation of Gram-positive bacteria through intestinal epithelial cells through upregulation of the platelet-activating factor receptor [53]. Differences in innate immunity responses against invading gram-negative and gram-positive pathogens were recently highlighted in a *C. elegans* model. Whether HIF upregulation in epithelial cells is protective or damaging for bacterial defence mechanisms, could depend on the type of bacterial pathogen (Gram-positive versus Gram-negative) and on the epithelial cell type involved. It is intriguing to speculate that stimulation of hypoxia dependent signalling pathways elicits protective mechanisms in pulmonary epithelial cells against invasion of pathogens. Empowering host defences could represent a novel strategy against bacterial pathogens which are challenging us with continuously increasing antimicrobial resistance.

In summary, we have shown that hypoxia or pharmacologic hydroxylase inhibition decreases internalization of *P. aeruginosa* into epithelial cells, attenuating cytotoxicity of *P. aeruginosa* infection. Decreased internalization was not *P. aeruginosa* strain specific and applied to the cystic fibrosis invasive pathogen *B. cenocepacia* as well. Decreased internalization in hypoxia was independent from NF-κB activity and CFTR expression. Both HIF2-α and RhoA are epithelial cell regulatory factors participating in this effect. In addition DMOG significantly improved survival in a murine *P. aeruginosa* pneumonia model.

## Supporting Information

Figure S1 **Internalization of** ***E. coli*** **in normoxia, hypoxia and in normoxia with cells pre-incubated in hypoxia.** Antibiotic exclusion assays were performed with *E. coli* in A549 cells under normoxic and hypoxic conditions or in normoxia after hypoxic pre-incubation. Data are shown as % of intracellular normoxic *P. aeruginosa* and represent mean ± SEM of 3 individual experiments.
(TIF)

Figure S2 **Enhanced NF-κB activity in intestinal epithelial cells in response to** ***P. aeruginosa*** **under hypoxic conditions.** (A) Caco2 cells were incubated with heat inactivated *P. aeruginosa* in normoxia (N) and hypoxia (H). A representative immunoblot out of 3 independent experiments is shown.
(TIF)

Figure S3 **Stress fibre formation is increased in hypoxia and attenuated by inhibiting RhoA or HIF-2α.** HepG2 cells (wt, CV, shHIF1α, shHIF2α) were incubated in normoxia or hypoxia for 24 h, additionally wt cells were treated with 10 µM Y27632. The cells were stained for F-actin with Phalloidin. Green: Phalloidin, blue: Höchst, bar: 10 µm.
(TIFF)
