# Supplementary figures and images for: Hypoxia Modulates Infection of Epithelial Cells by Pseudomonas aeruginosa
Source: PLoS One. 2013 Feb 13;8(2):e56491. doi: 10.1371/journal.pone.0056491 (PMC3572047; doi:10.1371/journal.pone.0056491)

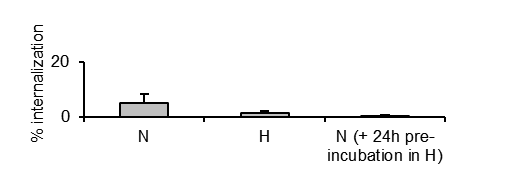

Supplement: Figure S1 — Internalization of E. coli in normoxia, hypoxia and in normoxia with cells pre-incubated in hypoxia. Antibiotic exclusion assays were performed with E. coli in A549 cells under normoxic and hypoxic conditions or in normoxia after hypoxic pre-incubation. Data are shown as % of intracellular normoxic P. aeruginosa and represent mean ± SEM of 3 individual experiments. (TIF) [file pone.0056491.s001.tif]

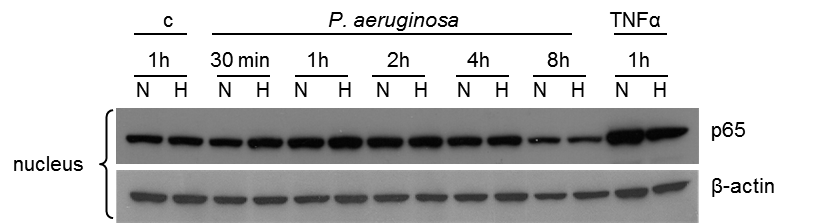

Supplement: Figure S2 — Enhanced NF-κB activity in intestinal epithelial cells in response to P. aeruginosa under hypoxic conditions. (A) Caco2 cells were incubated with heat inactivated P. aeruginosa in normoxia (N) and hypoxia (H). A representative immunoblot out of 3 independent experiments is shown. (TIF) [file pone.0056491.s002.tif]

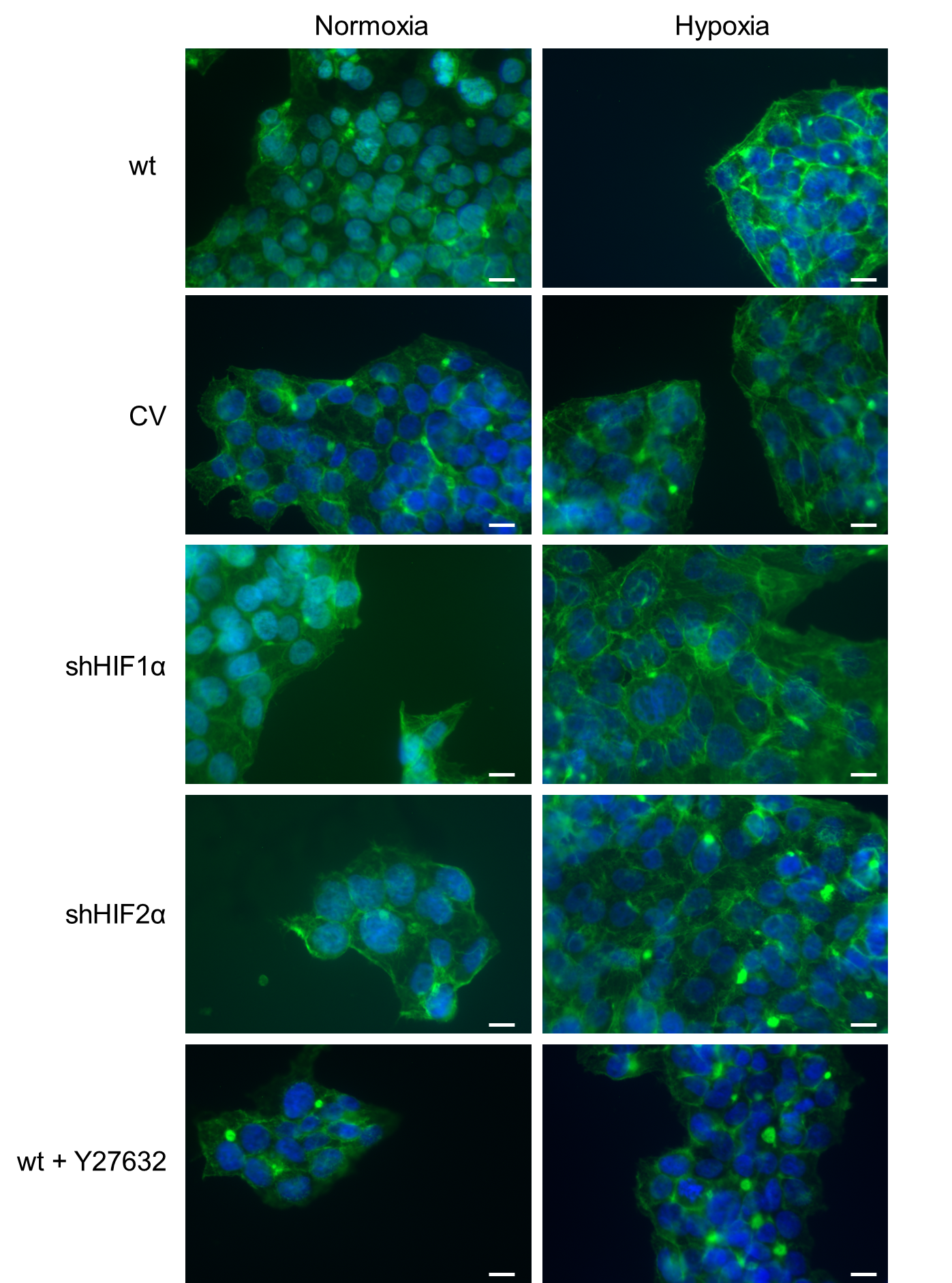

Supplement: Figure S3 — Stress fibre formation is increased in hypoxia and attenuated by inhibiting RhoA or HIF-2α. HepG2 cells (wt, CV, shHIF1α, shHIF2α) were incubated in normoxia or hypoxia for 24 h, additionally wt cells were treated with 10 µM Y27632. The cells were stained for F-actin with Phalloidin. Green: Phalloidin, blue: Höchst, bar: 10 µm. (TIFF) [file pone.0056491.s003.tiff]
